# Displaced proximal humeral fractures: an Indian experience with locking plates

**DOI:** 10.1186/1749-799X-5-60

**Published:** 2010-08-23

**Authors:** Sameer Aggarwal, Kamal Bali, Mandeep S Dhillon, Vishal Kumar, Aditya K Mootha

**Affiliations:** 1 Deptartment of Orthopaedics, PGIMER, Chandigarh Postgraduate Institute of Medical Education and Research, Sector 12, Chandigarh - 160 012, India

## Abstract

**Background:**

The treatment of displaced proximal humerus fractures, especially in elderly, remains controversial. The objective of this study was to evaluate functional outcome of locking plate used for fixation of these fractures after open reduction. We also attempted to evaluate the complications and predictors of loss of fixation for such an implant.

**Methods:**

Over two and a half years, 56 patients with an acute proximal humerus fracture were managed with locking plate osteosynthesis. 47 of these patients who completed a minimum follow up of 1 year were evaluated using Constant score calculation. Statistical analysis was done using SPSS 16 and a p value of less than 0.05 was taken as statistically significant.

**Results:**

The average follow up period was around 21.5 months. Outcomes were excellent in 17%, good in 38.5%, moderate in 34% while poor in 10.5%. The Constant score was poorer for AO-OTA type 3 fractures as compared to other types. The scores were also inferior for older patients (> 65 years old). Complications included screw perforation of head, AVN, subacromial impingement, loss of fixation, axillary nerve palsy and infection. A varus malalignment was found to be a strong predictor of loss of fixation.

**Conclusion:**

Locking plate osteosynthesis leads to satisfactory functional outcomes in all the patients. Results are better than non locking plates in osteoporotic fractures of the elderly. However the surgery has steep learning curve and various complications could be associated with its use. Nevertheless we believe that a strict adherence to the principles of locking plate use can ensure good result in such challenging fractures.

## Background

Proximal humeral fractures account for almost 4 to 5% of all fractures[1,2]. These fractures have a dual age distribution occuring either in young people following high energy trauma or in those older than 50 years with low velocity injuries like simple fall [3]. Three fourths of the fractures occur in older individuals with an occurrence three times more often in women than in men [3,4]. Most of the proximal humeral fractures are nondisplaced or minimally displaced and stable. These can be treated nonoperatively successfully with early rehabilitation[5-7]. But severely displaced and comminuted fractures warrant surgical management for optimum shoulder function. Surgeons should be familiar with the different treatment options available, including recent advances in the management of periarticular fractures [8-14] and in locking plate technology [11,15] which are particularly relevant to the care of these fractures[10,16-18].

Traditional treatment techniques include open reduction and internal fixation with proximal humeral plates, hemiarthroplasty, and percutaneous or minimally invasive techniques such as pinning, screw osteosynthesis, and the use of intramedullary nails[12-14,19-24]. All these techniques have been associated with various complications including implant failure, loss of reduction, nonunion or malunion of the fracture, impingement syndrome, and osteonecrosis of the humeral head[13,25-27]. Locking plate technology has been developed as a solution to the problems encountered during conventional plating to treat fractures in osteoporotic bone particularly with metaphyseal comminution. The key to this technology is fixed angle relationship between the screws and plate. The threaded screw heads are locked into the threaded plate holes to prevent screw toggle, slide and pull out, thus diminishing the possibility of primary or secondary loss of reduction. Even biomechanical analysis studies have showed the superiority of such a fixation when compared to a blade plate fixation[28].

However till now very limited prospective studies have been done describing the functional outcome and complications following locking plate fixation of proximal humeral fractures [9,19,29,30]. There may be special technical requirements for the success of such a plate which need to be defined. Thus the objective of our study was to determine the efficacy of proximal humerus interlocking system (PHILOS), to evaluate the complications and to identify the predictors of loss of fixation of such an implant.

## Materials and methods

This prospective study included a series of 56 patients operated between September 2006 and Feb 2009 with a proximal humerus locking plate for displaced fracture of proximal humerus.

Inclusion criteria included:

1. Closed two part fracture with a major displacement of the humeral diaphysis or three or four part fracture having a tuberosity displacement enough to cause a significant subacromial impingement.

2. Patients operated within 10 days of injury.

3. Patients with a minimum follow up period of 1 year.

Exclusion criteria included:

1. Skeletally immature patients

2. Patients with open fractures,

3. Pathological fractures,

4. Patients with distal neurovascular deficit,

5. Patients with nonunions, malunions or delay in surgery(>10 days),

6. Displaced three or four part fractures with significant bone loss(as seen on CT scan) suggesting insufficient screw purchase and thus treated by humeral arthroplasty.

7. Concomitant ipsilateral fracture of distal humerus or elbow joint,

8. Polytrauma patients with an Injury Severity Score > 16

All proximal humeral fractures met the indications for the operative treatment as outlined by Neer [31] i.e. an angulation of articular surface of more than 45 degrees, a displacement between the major fracture fragments more than 1 cm or a fracture with valgus impaction [32].

Preoperative true AP, scapular, lateral and axillary X rays along with CT scans of the area were reviewed by two of the specialist orthopedic surgeons to define fracture type and outline the plan of surgery. Fracture patterns were classified according AO/OTA system[33] and the Neer classification[34].

Surgery was performed in supine postion on a radiolucent table using the deltopectoral approach. Fracture fragments were reduced without stripping periosteum to best possible anatomical position and reduction was held with Kirschner wires. Reduction was assessed under image intensifier. Definitive fixation with proximal humerus locking plate was done with plate positioned at least 5 mm distal to the upper end of of the greater tuberosity and at least 2 mm posterior to the bicipital groove thus sparing the tendon of long head of biceps. Plate was first fixed with K-wires through the holes. Then with maintenance of prior achieved reduction, multidirectional screws were used to fix proximal fragments. Rotator cuff, capsule and subscapularis muscle tears/avulsions were repaired meticulously. Tuberosities, whenever found fractured, were fixed to the plate applying tension band principle and using nonabsorbable sutures. The decision regarding the use of locking or the cortical screws for plate fixation to the humeral shaft was left to the discretion of the operating surgeon with locking screws being preferred for the older patients with suspected osteoporotic bones.

The post operative rehabilitation protocol included immediate passive and active assisted range of motion exercises up to 60 degrees of abduction and elevation with no forced external rotation for 6 weeks. Full ROM with active exercises was started at 6 weeks.

Patients were followed up on OPD basis at two weeks postoperatively, then monthly for 6 months, 3 monthly till the end of 1^st ^year and yearly thereafter. At every follow up visit standard AP and axillary radiographs were obtained and thorough clinical assessment done. Anticipated postoperative complications included loss of reduction, fragment displacement, major varus or valgus deformation, head necrosis or implant-related problems (screw perforation, screw loosening or backing out, plate pullout, or breakage), and surgical and other general complications such as wound infection or soft-tissue problems (rotator cuff lesions, adhesions, frozen shoulders, impingement, and nerve lesions). Functional outcome was assessed using the Constant score [35]. The Constant score was graded as poor (0-55 points), moderate (56-70), good (71-85) or excellent (86-100). To access for the potential effect of learning curve on the outcome, we arbitrarily divided the patients into two categories; patients operated by us in or before December 2007 and patients operated by us in or after January 2008.

Statistical analysis was done using SPSS version 16. A p-value of less than 0.05 was taken as statistically significant while a p-value between 0.05 to 0.1 was taken as trend towards significance.

## Results

During follow up, 3 patients died of unrelated pathologies while 6 patients were lost to follow up. Thus a total of 47 patients who completed the follow up were evaluated in our study. There were 27 males while 20 females. Mean age of the patients 58.51 years (23-81 years). The average follow up period was 21.49 months (12-38 months). In our study, out of a total of 47 patients, 27 were found to be older than 65 years of age suggesting a strong relation of proximal humerus with age related osteoporosis. Further, males 65 years or younger were more likely to sustain high energy fractures (n = 19/20, 95%) and female 65 years and older were more likely to sustain low energy fracture (n = 19/27, 70.37%) and this result was found to be statistically significant (p = 0.000). Falls accounted for 55% of fractures, road side accidents 42.5% and 1 fracture was caused by seizure. Table 1 shows the distribution of fractures according to age groups while table 2 shows the distribution of fractures according to Neers and AO-OTA classification.

**Table 1:** Distribution of fracture types according to age groups

	>65 years old	<65 years old
AO-OTA Type A	8	3

AO-OTA Type B	11	11

AO-OTA Type C	8	6

Total	27	20

**Table 2:** Distribution of fracture types according to Neer's classification and AO/OTA classification

Neer type	n	AO/OTA type	subtype	n	subtotal
2 part	13	Type A	2.2	3	11
			3.1	3	
			3.2	5	

3 part	23	Type B	1.3	5	22
			2.2	3	
			2.3	6	
			3.1	5	
			3.3	3	

4 part	10	Type C	3.2	4	14
			3.3	10	

Fracture dislocation	1				

Head splitting fracture	0				

Total	47				47

All fractures united with an average union time of 20 (16-25) weeks. Table 3 and table 4 shows Constant scores of the patients at the final follow up visit according to fracture types and age respectively.

**Table 3:** Constant score at last follow up according to fracture type (AO-OTA type)

Type A (n = 11)	Type B (n = 22)	Type C (n = 14)	All types (n = 47)	P value
77.54 ± 10.21 (64-92)	73.22 ± 10.67 (52-92)	66.00 ± 12.61 (42-86)	72.08 ± 11.77 (42-92)	0.039*

* significant

**Table 4:** Constant score at follow up visits according to the age of patient

>65 years old(n = 27)	<65 years old(n = 20)	All (n = 47)	P value
68.51 + 11.44 (42-88)	76.90 + 10.67 (52-92)	72.08 ± 11.77 (42-92)	0.013*

* significant

We found that patients with Type A fractures had the highest Constant scores while patients with Type C had the lowest Constant scores and these results were found to be statistically significant (p value 0.039). The Constant scores were found to be higher in younger patients as compared to older patients and this result was also found to be statistically significant (p value = 0.12). Overall the functional outcome was found to be moderate to excellent in 90% of our patients. however almost 10% patients had poor outcome. These results are shown in table 5. Various complications seen in our study have been shown in table 6.

**Table 5:** Functional outcome on the basis of Constant score at the last follow up visit

	Excellent	Good	Moderate	Poor	Total
Total	8	18	16	5	47

AO-OTA Types (A/B/C)	4/3/1	3/10/5	4/7/5	0/2/3	11/22/14

Age (<65 yrs/>65 yrs)	5/3	9/9	5/11	1/4	20/27

Percentage	17.02%	38.30%	34.04%	10.64%	100%

**Table 6:** Various complications seen in our study.

Complications	No. of patients	Early cases*	Late cases**
Failure of fixation or screw back out	5	3	2

Primary screw perforation of humeral head	6	5	1

Symptomatic AVN humeral head	2	0	2

Subacromial impingement	5	4	1

Non-union/Delayed union	0	0	0

Axillary nerve palsy	2	1	1

Deep wound infection	3	2	1

Superficial wound infection	6	4	2

* Before Dec 2007

** After Jan 2008

A varus head shaft axis on immediate postoperative X-rays and at last follow up visit was found to be a strong predictor of poor Constant score. However a valgus alignment was found to have no effect on the final Constant score. This result is highlighted in table 7

**Table 7:** Comparison of head shaft axis with mean Constant score at follow up

	Immediate postoperative (no.)	Last follow up (no.)	Constant score at last follow up
Normal	38	37	73.05 ± 12.01

Major Varus (<120°)	2	5	63.60 ± 12.44

Major Valgus (>160°)	7	5	73.40 ± 6.38

We also found that patients operated by us earlier (before Dec 2007) had somewhat inferior Constant scores at follow up as compared to the patients operated by us later on (after Jan 2008). A higher number of complications were also seen in the patients operated by us earlier. These results are highlighted in table 6 and table 8.

**Table 8:** Comparison of the cases operated by us earlier (before Dec 2007) as compared to the cases done later (after Jan 2008).

	Cases done earlier	Cases done later on	p-value
Number	19	28	

AO-OTA types (A/B/C)	7/5/7	5/17/6	

Mean Constant score	68.31 ± 13.47	74.64 ± 9.92	0.082**

Number of complications (29)	19	10	

** trend towards significance

## Discussion

Displaced proximal humeral fractures have always posed a challenge to treatment especially when associated with osteoporosis and communition. Such fractures usually require operative intervention to ensure correct positioning of the fracture fragments and to allow early mobilization. Osteoporosis predisposes to low energy fractures having a complex pattern [36] and difficult fixation owing to poor screw purchase [37,38]. Rate of failure of fixation is also high.

Various techniques [14,19,21,22,25] have been utilized for the treatment of these fractures and include intramedullary nails, plate osteosynthesis, tension band wiring, percutaneous K-wire fixation and hemiarthroplasty. Varying outcomes have been reported with plate osteosynthesis for proximal humerus fractures [10,13,22,25]. Whereas such fractures in young have uniformly good results with plate and screw fixation, results in osteoporotic fractures of elderly patients are often poor.

Esser[39] reported excellent results in 22 out of his 26 patients of three part and four part fractures of proximal humerus treated with a modified clover leaf plate. Wijgman et al[22] et al reported good to excellent results in 87% of their 60 patients with three or four part proximal humeral fractures operated with a T-buttress plate and cerclage wires. Paavolainen et al[40] reported satisfactory results in 74.2% of their 41 patients with severe proximal humerus fractures treated with plate and screw devices. However all these authors found poor results in 4 part fractures and recommended a prosthetic replacement in such patients.

The recent evolution of locking plate technology for proximal humerus fractures seems to have revolutionized the management of these fractures. However there have been very limited prospective studies investigating the results of locking plates for open reduction and internal fixation of proximal humeral fractures[9,19,29,30,41]. Most of these studies have reported good functional outcomes and recommended the use of locking plates for proximal humerus fractures especially in elderly patients with poor bone quality.

The results of our prospective study showed good or excellent outcomes in around 56% of our patients. These results were somehow inferior to those reported in the western literature. Patients operated by us earlier when the locking plate principles had just been introduced showed somewhat inferior results as compared to those operated later and this result showed a trend towards significance (p = 0.082) on Chi square analysis. Also a higher number of complications were seen in the patients operated by us earlier. This leads us to believe that application of locking plate technology for proximal humerus fractures has a steep learning curve and appropriate surgical technique is very important for achieve good functional outcome.

We also found inferior results with AO-OTA type 3 fractures which is expected as these fractures are more complex and open reduction and internal fixation is tougher. The results were also inferior in patients with age older than 65 years. Neverthless our results in older age patients are better than those of traditional plates used in such osteoporotic fractures[22,39,40]. We, thus believe, that a locking plate device for proximal humerus fractures gives a satisfactory outcome in most of the patients including those with old the age and poor bone density.

**Figure 1:**
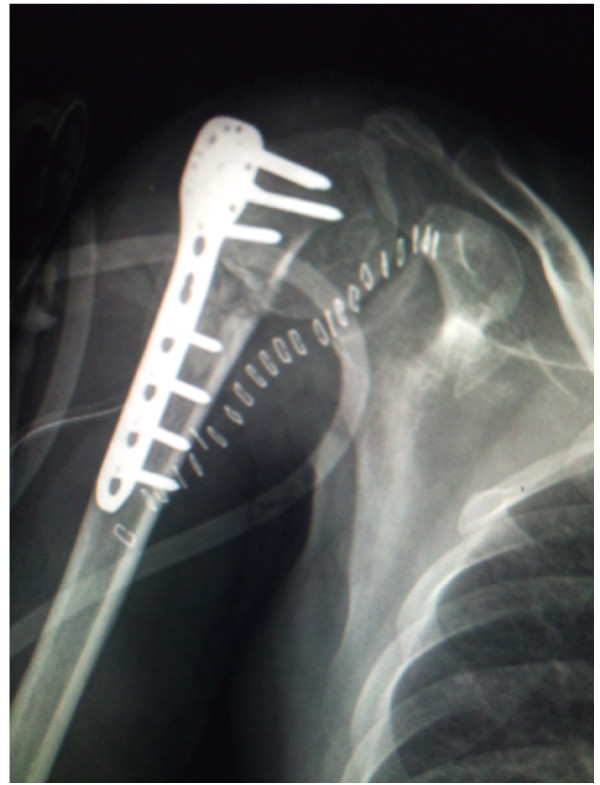
**Immediate post operative X-ray in a patient showing varus collapse and plate pull out**.

As it was a large cohort of patients, various complications were encountered by us. Varus malalignment (head shaft angel < 120°) was noted immediately postoperatively in 2 of our patients (Fig 1), one each in C2 and and C3 group. At further follow up, 3 more patients showed varus collapse. Subsequent loss of reduction was seen in all five of these patients. Three of these patients underwent revision surgery with implant removal and new proximal humerus locking plate. Other two were operated by shoulder hemiarthroplasty later on considering the highly comminuted and intra-articular nature of the fracture. None of the patients with a neutral or valgus alignment had a loss of fixation at long term follow up. We thus found that a varus malalignment was a strong predictor of loss of fixation.

**Figure 2:**
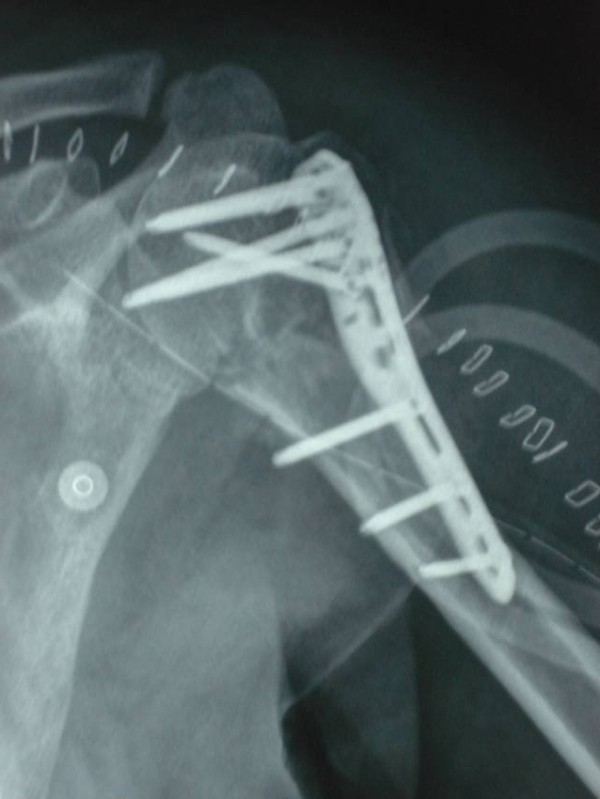
Immediate post operative X-ray in a patient showing primary screw perforation.

Six patients were found to have primary screw perforation (Fig 2) of the humeral head that was unrecognized during the surgery. An early implant removal was done in two of these patients while four of the patients underwent a repeat surgery to exchange the screws for shorter screws.

Symptomatic humeral head AVN was noted in two patients with C.3.3 fractures at follow up visits. Both of them were later operated with hemiarthroplasty and the result was found to be good.

We observed subacromial impingement to start with in 5 of our patients. This was thought to be a result of too far cranial positioning of the plate. However with time all of these patients improved and plate removal was done in only 2 of these patients after the fracture had united.

No case of non union or delayed union was seen. There were 2 cases of axillary nerve palsy. However no intervention was required in any of these and both the patients improved within 1 year of follow up. Deep wound infection was seen in 3 patients. Two of these settled after debridement surgeries. Implant removal was done in one of the patients who was reoperated later; repeat plating being done 4 months after the infection had settled. However superficial wound infection, not requiring a formal debridement, was found to be common, seen in 6 of our patients. All these patients subsequently settled with an extended course of IV antibiotics and local wound treatment.

**Figure 3:**
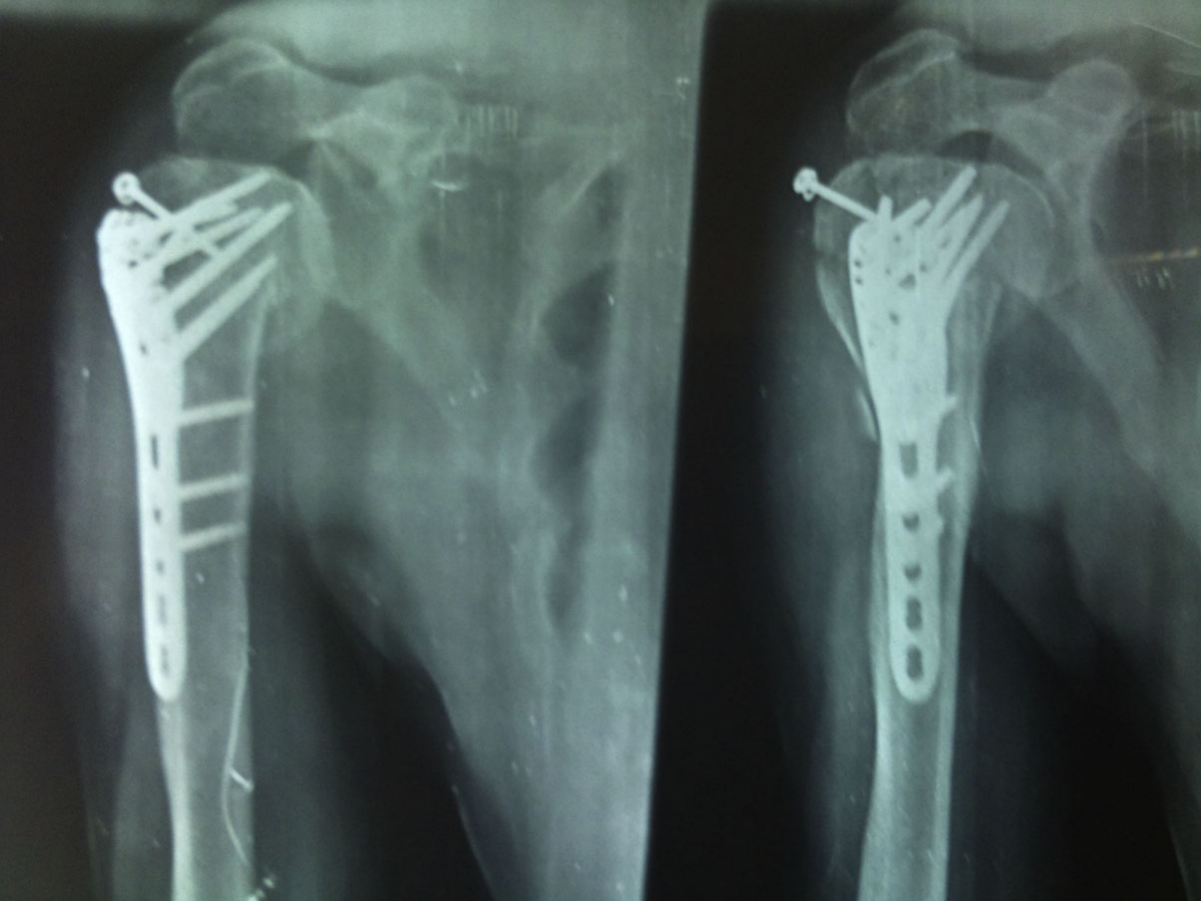
Varus collapse in a patient on follow up X-rays, both AP and lateral.

In our present study, proximal humerus locking plate has shown promising result in displaced and comminuted proximal humeral fractures. Loss of reduction occurred in 10% of patients(5 patients) after implant loosening in proximal fragments. Varus malreduction (Fig 3) has been found to be a predictor of such of reduction and must be avoided intraoperatively at every cost.

Most of the complications in our series occurred during our initial experience (table 5). Out of the 6 patients with screw perforations into the joint, 5 happened in our earlier cases. As our experience increased, we realized that the best way to avoid this was to get confirmatory radiographs throughout the arc of rotation (maximum internal to maximum external rotation) after the hole has been drilled (with drill bit in situ) to get the exact length of the screw. We preferred to put a smaller sized screw whenever the length measured fell between two screw sizes. Impingement occurred in 5 of our cases and again 4 of them were in the initial experience. We feel that the best way to avoid superior placement of the plate is to provisionally fix the plate with k-wires through the superior most holes of plate (small holes meant for k-wires), check under fluoroscopy throughout the arc of abduction and then proceed further. All the screw pull outs occurred in osteoporotic cases. We personally feel that the best way to tackle this problem is to put as many screws in the head as possible; however we did not evaluate this factor as the number of osteoporotic cases was too small to be analyzed. Augmentation with PMMA cement is an option and Matsuda et al [42] have reported a series of 5 such cases. However we do not have any personal experience with cement augmentation. Most of the infections especially superficial ones had also occurred during our initial phase and we feel that this was mainly due to poor soft tissue handling and raising of excessive skin flaps. As our surgical technique evolved, infectious complications were found to occur less frequently.

A potential limitation of our study was the absence of a control group treated by a different modality. Thus we cannot actually determine if any other method of treatment would have led to different results. Nevertheless our results are better than those of the previous studies in which plate osteosynthesis other than locking plate has been used[22,39,40]. Also the prospective design of our study, the large sample size (47 patients) and a decent average follow up period (21.5 months) adds strength to our study.

In a recently published study protocol by Handoll et al [43], the authors aim to undertake a multicentric randomized control trial to evaluate the efficacy and cost effectiveness of surgical versus standard nonsurgical treatment for adults with an acute closed displaced fracture of the proximal humerus with involvement of the surgical neck. Probably the outcome of this study will further add to our existing knowledge about management of these complex fractures of proximal humerus.

To conclude, we believe that a locking plate for the treatment of proximal humerus fractures uniformly leads to a satisfactory functional outcome over long term follow up in most of the patients. Although the results are poorer in old aged individuals with osteoporosis, they are nevertheless better than those achieved with non locking plates. The AO-OTA type 3 fractures have poorer results as compared to type 1 or type 2 fractures. However the results in type 3 fracture are good enough to recommend open reduction and internal fixation with locking plates in these patients. A varus malalignment was found to be a strong predictor of loss of fixation and should be avoided if possible. The surgery carries a steep learning curve and various complications could be associated with it. However, proper use of locking plate principles and a meticulous soft tissue repair with aggressive post operative rehabilitation go a long way in ensuring a satisfactory functional outcome.

## Competing interests

The authors declare that they have no competing interests.

## Authors' contributions

KB reviewed the literature and wrote the paper. SA and MSD were main operating surgeons in the whole series and critically reviewed the paper. KB, VK and AKM maintained all the records of the patients and followed them. All the authors read and approved the final manuscript.
